# Abciximab in rescue therapy of thromboembolic events during endovascular treatment of cerebral aneurysms: single center experience of 49 cases

**DOI:** 10.3389/fneur.2025.1573247

**Published:** 2025-06-25

**Authors:** Lukas Lenhart, Valentin Ladenhauf, Verena Rass, Stephanie A. Treichl, Ronny Beer, Christian F. Freyschlag, Elke R. Gizewski, Astrid E. Grams

**Affiliations:** ^1^Department of Radiology, Medical University of Innsbruck, Innsbruck, Austria; ^2^Department of Neurology, Medical University of Innsbruck, Innsbruck, Austria; ^3^Department of Neurosurgery, Medical University of Innsbruck, Innsbruck, Austria

**Keywords:** abciximab, endovascular neuroradiology, intracranial aneurysm, thromboembolic complication, cerebral infarction, intracerebral hemorrhage

## Abstract

**Background:**

The aim of this study was to assess the safety and effectiveness of abciximab (ReoPro®), a glycoprotein-IIb/IIIa receptor antagonist, in the management of thromboembolic complications during endovascular treatment of intracranial aneurysms.

**Methods:**

From an inhouse data base of 218 coil embolisations in 178 patients with cerebral aneurysms (median age 57 years, 72% females)–among them 53 interventions with subarachnoid hemorrhage (SAH) requiring acute endovascular treatment, and 165 with elective endovascular treatment of unruptured aneurysms - we retrospectively reviewed 53 interventions that received abciximab during endovascular treatment, and diffusion-weighted imaging after endovascular treatment (median 7 days after intervention, range 5 to 9 days). Four subgroups were identified, (i) SAH patients with abciximab (SAH-Ab, *n* = 18), (ii) SAH patients without abciximab (SAH-no, *n* = 35), (iii) elective patients with abciximab (El-Ab, *n* = 31), and (iv) elective patients without abciximab (El-no, *n* = 134) treatment. Angiographic success of clot dissolution, post-interventional occurrence of intracranial hemorrhages and the modified Rankin Scale (mRS) pre and 6 months after endovascular treatment were recorded. Brain tissue infarction-load was categorized as: no, punctual (<500 mm^2^), small (500–1,000 mm^2^) and large (>1,000 mm^2^) infarctions.

**Results:**

Complete and partial angiographic success was achieved in 94% of cases. No significant differences in infarction-loads were found between SAH-Ab and SAH-no, or El-Ab and El-no subgroups. Abciximab use was not associated with increased infarction-loads or worse mRS scores. Hunt & Hess score at admission significantly predicted both infarction-loads (*β* = 0.38, *p* = 0.02) and 6-month mRS (*β* = 0.75, *p* < 0.001) in SAH patients. Two patients experienced post-interventional hemorrhages: one small bleed near the extraventricular drain in in the SAH-Ab group, and one large hemorrhage due to wire perforation in the El-Ab group. No fatal hemorrhages occurred following abciximab administration.

**Discussion:**

Abciximab was safe and seemed effective for the management of thromboembolic complications during endovascular treatment of intracranial aneurysms, without increasing infarct burden or compromising functional outcomes in affected patients.

## 1 Introduction

Despite prevention strategies during endovascular therapy (ET) of intracranial aneurysms, periprocedural thrombembolic complications (TC) are estimated to occur at a rate of 9.3% (2–15.5%), as reported in a study comprising 515 interventions (1) and recited in two recent meta-analyses (2, 3). A common rescue strategy, in case of TC, is intra-arterial (IA) and/or intra-venous (IV) administration of abciximab, to induce clot dissolution. Abciximab is a glycoprotein-IIb/IIIa inhibitor that blocks over 80% of receptors on platelet surfaces, thereby inhibiting their fibrinogen-mediated aggregation (2). The effect occurs within 2 to 10 min after the application (4, 5). Despite a short half-life of approximately 30 min, it may take over 48 h for plateled activity to return to normal (6). Intracranial hemorrhage is a known adverse event following administration of abciximab, but IA administration occurs at low doses and therefore may have a lower risk of bleeding (7). Optimal dosage of IA and IV abciximab for TC during ET of intracranial aneurysms is not consistent, since recommendations only exist for cardialvascular indications.

The aim of the present study was to compare the presence and amount of cerebral infarctions, clinical outcomes and bleeding rates in patients with and without periprocedural IA abciximab application in elective and acute ET of intracranial aneurysms. The control groups without TC were assumed to display reasonable controls, with “inevitable” infarctions, which are possible even without visible clots. The hypothesis was that patients who received IA abciximab have similar infarction rates and extents, clinical outcomes, and bleeding rates as patients who did not receive the drug.

## 2 Materials and methods

### 2.1 Patient population and endovascular interventions

Between the years 2012 and 2018, 305 patients with intracranial aneurysms admitted to the neurosurgical or neurological intensive care unit (ICU) of a tertiary hospital and 366 aneurysms were treated endovascularly by the neuroradiology department of the Medical University of Innsbruck. Patients with available MRI including diffusion-weighted imaginag (DWI) within 7 days after the intervention were included in the study. The final sample comprised 178 patients (128 women and 50 men; 3 to 76 years old; mean age 57 years) with 218 interventions eligible for a retrospective analysis. Of the interventions, 165 were elective procedures for incidental or previously treated aneurysms with reperfusion, while 53 were acute cases requiring emergency ET for SAH. Four subgroups were defined: SAH patients with abciximab (SAH-Ab), SAH patients without abciximab (SAH-no), elective patients with abciximab (El-Ab) and elective patients without abciximab (El-no) treatment.

In SAH patients, admission Hunt and Hess (H&H) grades, and additional parameters of the inpatient stay were extracted from electronic patient files. Delayed cerebral ischemia (DCI) was diagnosed in the setting of clinical deterioration with a new focal neurologic deficit, a decrease of greater than or equal to two points on the Glasgow Coma Scale, or a new infarct on the CT or MRI not attributable to other causes (8).

This retrospective study was conducted using anonymized patient data. Local institutional ethics committee approval was not required due to the retrospective nature of the study and the use of de-identified data. All procedures were carried out in accordance with the ethical standards of our institution and with the 1964 Helsinki Declaration and its later amendments.

### 2.2 Endovascular interventions and anticoagulant medication

The optimal treatment method for every aneurysm was determined interdisciplinarily by interventional neuroradiologists, neurologists and neurosurgeons. Interventions were performed using a biplane angiography system (PHILIPS Allura XPER FD20/20 biplane, Philips Medical Systems DMC GmbH, Hamburg, Germany). In 154 interventions only coils, in 64 cases intracranial stents or flow diverters were used with or without additional coils.

Pre-, peri- and post-interventional anticoagulant medication was standardized: for elective interventions pre-interventional loading with 100 mg of *Aspirine for 5 days* and additional 75 mg Clopidogrel for planned stent or flow diverter insertion. During the interventions, the guiding catheters were continuously flushed with solutions containing 1,000 mL sodium chloride 0.9% and 2000 IU heparine and 2 mg nimodipine (Nimotop®). In all patients, a post-interventional herparinisation for 24 h with an aimed activated partial thromboplastin time of 40–60 s was performed. In patients with implanted stents or flow diverters life-long 100 mg of *Aspirine and additionaly 75 mg of* Clopidogrel for six (stents) or 12 (flow-diverters) months was prescribed.

### 2.3 Abciximab

In 49 cases (18 SAH & 31 elective) abciximab was administered IA during the intervention with median dosage dose of 10.1 mg, range from 3 mg to 20 mg. A visible clot near the aneurysm neck or within the stent, defined as “proximal thrombus,” a delayed distal flow, defined as “distal thrombus,” seen on digital substraction angiography (DSA), were the criteria for the application. Abciximab was always administered after securing the aneurysm from hemorrhage. Mean arterial pressure (MAP) was raised medically in cases of TC with a target MAP of 90 mm/Hg during the intervention.

### 2.4 Post-interventional imaging analysis

**Figure 1:**
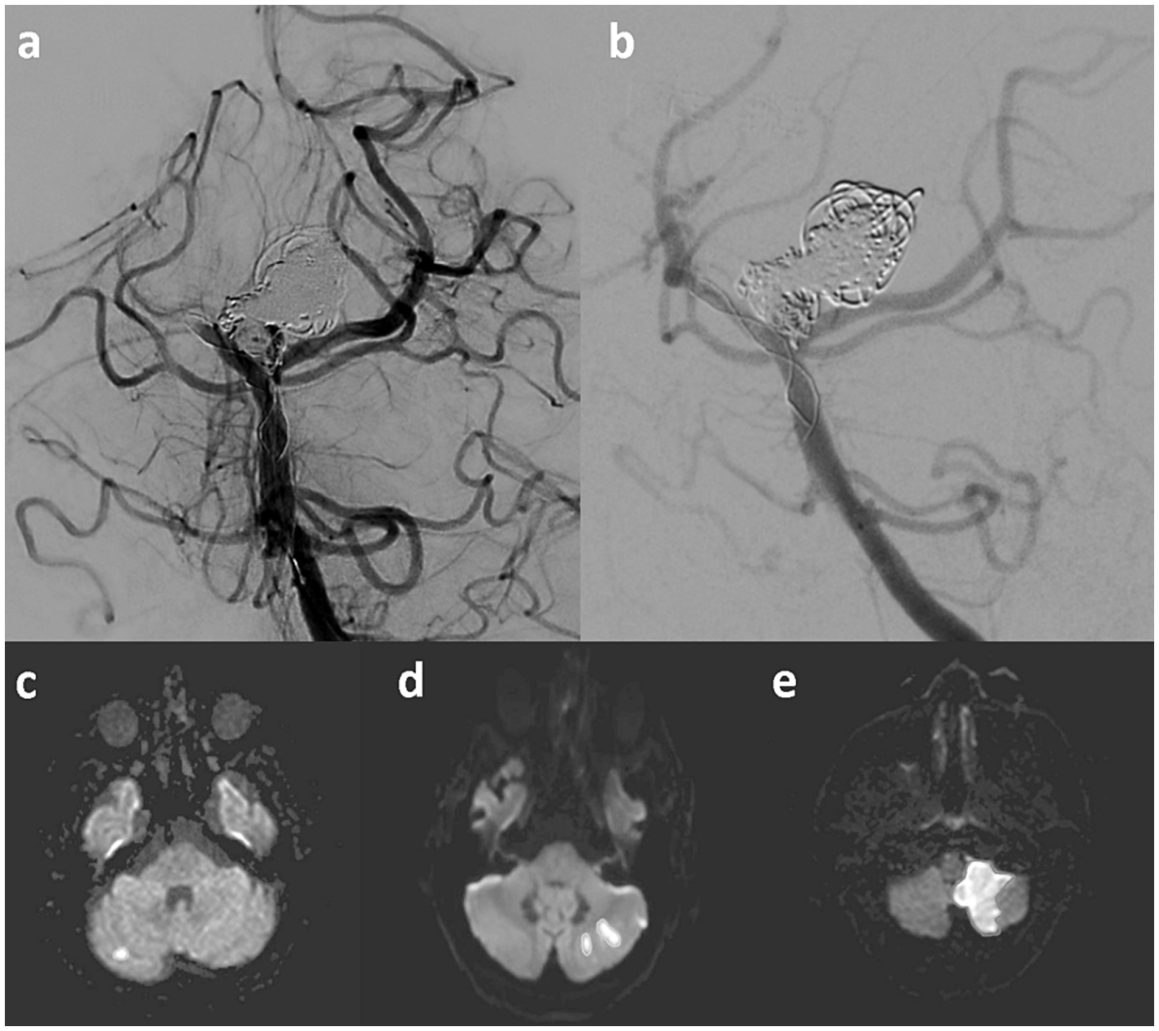
In the upper row an example of a stent-assisted coil occusion of a giant basilar artery aneurysm is given. During the intervention, a clot within the distal stent is occuring **(a)**, which completely dissolves after the administration of intra-arterial abciximab **(b)**. In the lower row representative examples of punctual **(c)**, small **(d)** and large **(e)** infarctions are displayed.

Angiographic success was evaluated using a three-point scale to classify the thrombus dissolution or distal flow restoration assessed in a control DSA 10 min after drug administration with “no = 1,” “partial = 2” or “complete = 3” dissolution, by an experienced interventional neuroradiologist (A. G.). It is worth noting that while the commonly used Thrombolysis in Cerebral Infarction (TICI) score was assessed, it was not included in this analysis. This decision was made because most cases were rated TICI 2b both before and after treatment, limiting its discriminatory value in this context. Instead, we found the three-point Likert scoring system to be more informative for our purposes. Figures 1a,b provide an example of a proximal clot with complete dissolution, illustrating the type of changes we aimed to capture.

The main radiological effectiveness outcome for the abciximab therapy was assessed with post-interventional MRI, performed 7 days after the intervention (range 5 to 9 days), according to a standardized protocol on 1.5 or 3 T MRI scanners. DWI was used to detect and quantifiy brain tissue infarction resulting from TC. Infarcted areas were measured manually in every slice with a suitable software (AGFA, IMPAX EE R20, Agfa HealthCare N. V., Mortsel, Belgium). The sum of all infarction areas, the infarction-load (IL), was calculated and subsequenty categorized as (1) no, (2) punctual (< 500 mm^2^), (3) small (500–1,000 mm^2^) and (4) large IL (> 1,000 mm^2^). Examples for each category are given in Figures 1c–e. Secondary intracranial hemorrhages within the first week were detected on post-interventional imaging and accurately interpreted as they represented an important safety parameter for the treatment with abciximab.

### 2.5 Clinical outcome

Changes of modified Rankin Scale (mRS), calculated by subtraction of the pre-interventional scores from the scores after 6 months, were used to monitor clinical outcomes. Nine SAH patients were transferred abroad after the acute phase and were therfore lost to follow up and excluded from mRS analysis.

### 2.6 Statistical analysis

Data were analyzed using IBM SPSS Statistics for Windows, Version 26.0 (IBM Corp., Armonk, NY, USA). Normality of data distribution was assessed using the Shapiro–Wilk test. As the data were not normally distributed, comparisons between two groups were performed using the Mann–Whitney U test. A multiple linear regression analysis was conducted to examine the relationship between postinterventional IL and mRS at 6 months and TC with abciximab administration. H&H score at admission, patient age, as well as the presence of secondary complications including vasospasm and DCI were entered as covariates. Statistical significance was determined using an F-test for the overall model and t-tests for individual predictors, with *α* = 0.05.

## 3 Results

Clinical and periprocedural characteristics, angiographic success, infarction quantification and clinical outcome data are outlined in Table 1. Of all aneurysms, 142 (65%) were in the anterior circulation, while 77 (35%) were in the posterior circulation. TE incidents during ET were observed in 49 of all treated aneurysms. In 34 of the 49 interventions, angiography showed a “proximal clot” and in 14 a “distal clot.”

**Table 1:** Clinical and periprocedural characteristics, angiographic success, infraction quantification and clinical outcome.

Variable	SAH (*n* = 53)			Elective (*n* = 165)		
	Abciximab (*n* = 18)	No abciximab (*n* = 35)	*p*	Abciximab (*n* = 31)	No abciximab (*n* = 134)	*p*
Age (years; mean ± SD)	49.9 (9.6)	55.2 (11.6)	>0.05	56.8 (10.2)	58.4 (11.6)	>0.05
Number (females; *n*)	12 (67%)	27 (77%)	>0.05	23 (74%)	97 (72%)	>0.05
Hunt & Hess Score (*n*)						
I	7 (39%)	11 (31%)	>0.05	–	–	
II	7 (39%)	5 (14%)	>0.05	–	–	
III	2 (11%)	8 (23%)	>0.05	–	–	
IV	2 (11%)	2 (6%)	>0.05	–	–	
V	0 (0%)	7 (20%)	>0.05	–	–	
Intervention						
Coils (*n*)	15 (83%)	34 (97%)	>0.05	27 (87%)	88 (66%)	>0.05
Coils and stent/ flow diverter (*n*)	3 (17%)	1 (3%)	>0.05	4 (13%)	46 (34%)	>0.05
MAP (mmHg; mean ± SD)	87 (8)	87 (9)	>0.05	82 (7)	80 (9)	>0.05
Need for re-intervention (*n*)	0	1 (3%)	>0.05	6 (19%)	40 (30%)	>0.05
Periprocedural complications and angiographic success rates						
Intracerebral haemorrhage	1	0		0	1	
Local thrombus (*n*)	13 (72%)	0		22 (71%)	0	
Peripheral thrombus (*n*)	5 (28%)	0		9 (29%)	0	
Drug amount (mg; mean ± SD)	8.5 (2)	0		11 (4.6)	0	
Angiographic success (mean ± SD)	2.4 (0.6)	–		2.3 (0.6)	–	
No success	1 (<1%)			2 (7%)		
Partial	9 (50%)			19 (61%)		
Complete	8 (49%)			10 (32%)		
Infarct categorization						
No infarctions (*n*)	6 (33%)	5 (14%)		14 (45%)	61 (46%)	
Punctual infarctions (*n*)	4 (22%)	10 (29%)		10 (32%)	67 (50%)	
Small infarctions (*n*)	4 (22%)	4 (11%)		0 (0%)	5 (4%)	
Large infarctions (*n*)	4 (22%)	16 (46%)		7 (23%)	1 (<1%)	
Infarction loads (mm^2^, mean ± SD)	24,286 (3042)	135,864 (9423)	>0.05	36,870 (4447)	48,168 (3026)	>0.05
60-day mRS (*n*)						
Good functional outcome at 6 months follow-up (mRS 0–2; n)	16 (89%)	17 (49%)	0.025	28 (90%)	127 (95%)	>0.05
mRS increases from pre to post intervention (mean ± SD)	0.6 (1)	2.4 (2.5)	0.044	0.5 (0.9)	0.6 (1.2)	>0.05

SAH, subarachnoid haemorrhage; n, number; SD, standard deviation; DCI, delayed cerebral ischemia; mRS, modified Rankin Scale; MAP, mean arterial pressure.

### 3.1 Anticoagulant medication

Doses of IA abciximab ranged from 3 mg to 20 mg with a mean dose of 10.07 mg (± 4.11 mg) per patient. One patient received an additional IV infusion of abciximab for 12 h with 0.125 μg/kg/min (total dose: 6.3 mg). In one patient abciximab was administered prophylactically in the setting of an acute intracranial stenting.

### 3.2 Angiographic success

After IA abciximab administration partial angiographic success was achieved in 28 (57%) and complete in 18 (37%) patients. In three interventions (6%) the therapy was not successful, one experienced a marked worsening with a complete angiographic vessel occlusion. No significant differences in the angiographic effect between SAH-Ab and El-Ab were found.

### 3.3 Infarction analysis

**Figure 2:**
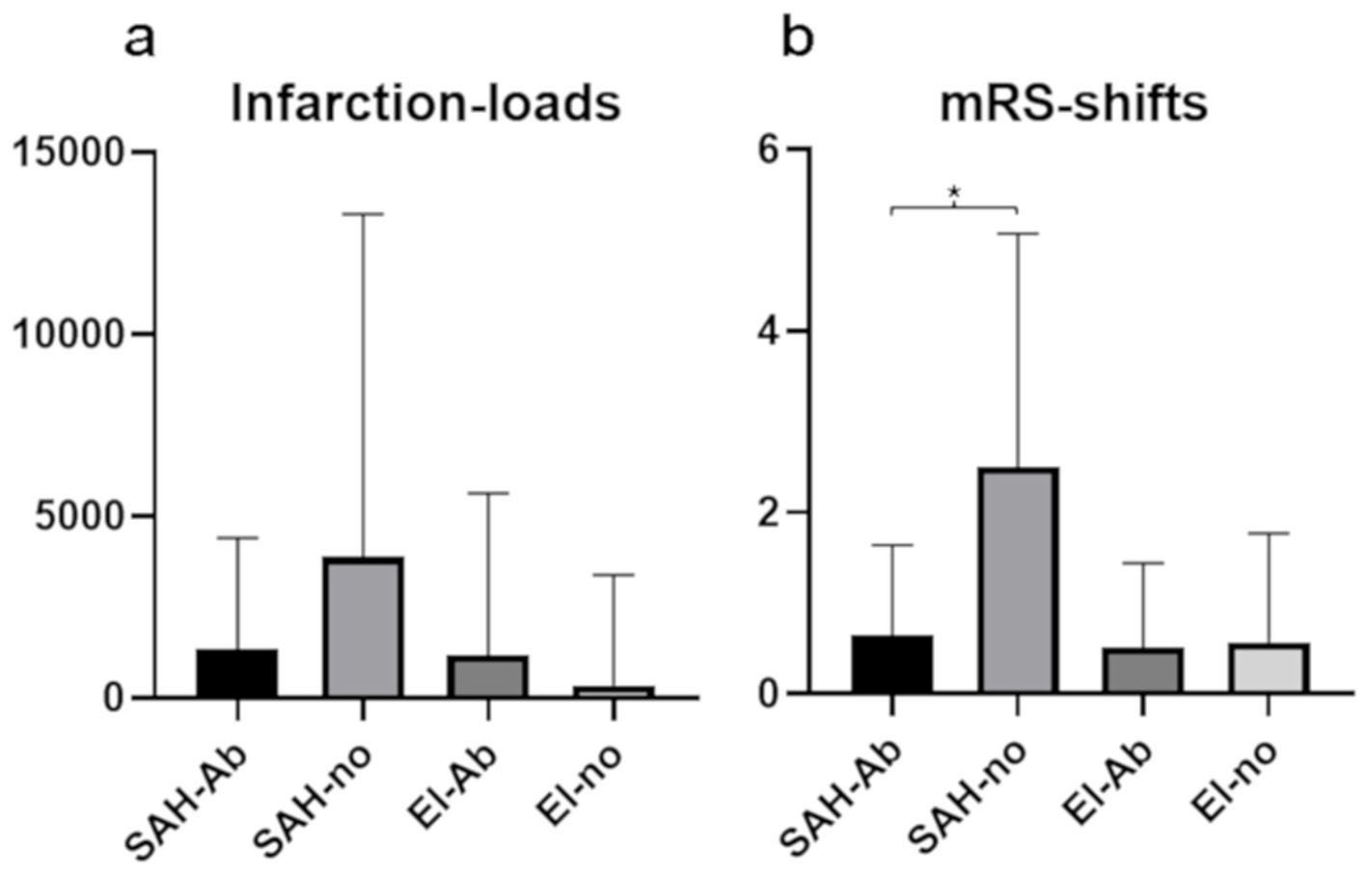
Mean values and standard deviations of infarction-loads **(a)** and modified Rankin Scale changes after 6 months **(b)** in the four subgroups. Significantly worse clinical outcome is seen in SAH patients without abciximab treatment (SAH-no), compared to SAH patients with abciximab administration (SAH-Ab).

No significant differences in IL were found between SAH-Ab and SAH-no, or El-Ab and El-no subgroups (Figure 2a). In SAH interventions, H&H score at admission was a significant predictor of IL (*β* = 0.38, t = 2.41, *p* = 0.02), indicating that higher H&H scores were associated with higher postinterventional IL, whereas no sigfnificant association was evident regarding TC and abciximab administration.

### 3.4 Clinical characteristics and outcomes

The distribution of H&H grades at admission was as follows: H&H 1: 18 patients, H&H 2: 14 patients, H&H 3: 10 patients, H&H 4: 4 patiens, and H&H 5: 7 patients (Tables 1, 2). Analysis revealed a statistically significant difference in mRS changes between SAH-Ab and SAH-no subgroups (*p* = 0.044), while no significant difference was observed between the elective groups (Figure 2b). Multiple regression analysis demonstrated that mRS at 6 months in SAH cases was predicted by H&H score at admission (*β* = 0.75, *t* = 8.67, *p* < 0.001), occurrence of DCI (*β* = 0.25, *t* = 2.45, *p* = 0.019), and age (*β* = 0.32, *t* = 3.87, *p* < 0.001). The occurrence of TC and abciximab administration was no significant predictor of functional outcome.

**Table 2:** Complications of further inpatient stay of the SAH interventions (*n* = 53).

Complication	Abciximab (*n* = 18)	No abciximab (*n* = 35)	*p*
Delayed cerebral ischemia (*n*)	2 (11%)	11 (31%)	>0.05
Vasospasm (*n*)	8 (44%)	18 (51%)	>0.05
Aneurysm rebleeding (*n*)	0	6 (17%)	>0.05
Duration of intubation (mean ± SD)	4.4 (5.1)	9.4 (11.5)	>0.05
Pneumonia (*n*)	5 (28%)	15 (43%)	>0.05
Ventriculitis (*n*)	1 (6%)	5 (14%)	>0.05

### 3.5 Intracerebral hemorrhage

Intracerebral hemorrhage was observed in one patient in the SAH-Ab group along the external cerebrospinal fluid drainage placed before the intervention. One further patient in the El-Ab group showed a large hemorrhage due to wire perforationon in post-interventional neuroimaging. *No fatal bleeding occurred due to abciximab administration.*

## 4 Discussion

Our findings suggest that abciximab is a safe and effective treatment for TC during ET of both acute and elective intracranial aneurysms, achieving complete or partial angiographic success in 94% of cases. In elective cases with TC treated with abciximab, IL and mRS scores were comparable to those without TC, supporting the hypothesis that abciximab may prevent or reduce infarctions resulting from TC during ET. Higher IL and mRS scores in SAH patients, who did not receive abciximab, were based on higher disease severity, rate of secondary complications and age. Post-interventional hemorrhages were very rare in all subgroups, underscoring the favorable safety profile of abciximab in managing TC during ET of intracranial aneurysms.

Intraprocedural angiographic success rates of partial or complete thrombus dissolution and low bleeding rates are largely in line with most previously published literature underlining the effectiveness and safety of abciximab as rescue strategy for TC during ET of intracranial aneurysms. A previous study comprising 30 patients with TC during flow diversion with pipeline embolization devices reported partial or complete recanalization after abciximab use in the majority of cases (77%), and infarctions (13%) or intracranial hemorrhages (7%) in few cases (9). Two studies in 42 patients with coiling (five stent-assisted) found infarctions in 31 and 33%, intracranial hemorrhages in 0 and 9.5% after rescue treatment with abciximab (1, 5). Another study examined the use of abciximab in 63 patients during or immediately following aneurysm coiling procedures, with one case also involving stenting. The results showed that 3.2% of patients experienced fatal intracranial hemorrhages, which occurred within areas of pre-existing subacute infarctions (10). In the present study, the frequency of visible ischemia in post-interventional imaging was more common, which can be explained by the use of DWI, and thus the sensitivity of detecting even punctate ischemias is significantly increased compared to previous studies that used CT scans. The occurrence of post-interventional hemorrhages was comparable lower to previous literature and maybe due to a more restrictive drug administration.

Additional evidence for the effectiveness of TC during ET of cerebral aneurysms comes from other studies that used abciximab for TC during aneurysm coiling. In a series of 13 patients complete recanalizations could be achieved in 92% (11). The peri-interventional IA use combined with the post-interventional IV administration over 12 h was proven efficient and safe in two further studies with 23 and 29 patients (12, 13). A study comprising 19 patients stated effectiveness of low doses IA abciximab, i.e., 10.5 mg ± 4.2, with no complications (7). Remaining relevant literature consists of one study in 13 patients with only IV treatment of abciximab (14), and several case series (15–18), predominantly reporting partial or complete angiographic success. In our study partial angiographic success with IA abciximab was achieved in 57% and complete success in 37% of drug administrations, respectively.

However, above mentioned beneficial effects regarding peri-interventional abciximab application must be contrasted with conflicting results and safety concerns of contradictory literature. One study involving 14 patients with ET in stroke therapy described the drug’s effectiveness as “less than optimal” with complete recanalization rates in 28.5% and at least partial recanalizations in 93%. This study further noted that clinical improvement was only associated with complete thromboembolic dissolution. In the same study, high complication rates (57%) were described including aneurysm recanalizations, distal thrombus migrations and one hemorrhagic complication (19).

The absence of significant differences in IL between abciximab-treated and non-treated groups suggests that abciximab use does not increase the risk of brain tissue damage. Importantly, the H&H score at admission emerged as a significant predictor of both IL and long-term functional outcomes, underscoring the critical role of initial clinical severity. While a statistically significant difference in mRS changes was observed between SAH subgroups treated with and without abciximab, this effect was not seen in elective cases. Multiple regression analysis further identified H&H score, patient age and the occurrence of DCI as significant predictors of six-month mRS scores in SAH cases. These findings emphasize the importance of initial clinical severity and early management of modifiable factors in determining patient outcomes (20, 21). Notably, TC treated with abciximab administration were not significant predictors of post-interventional IL and 6-months functional outcome. This is an important finding indicating that TC during ET may not impact negatively on long-term prognosis when treated with abciximab.

When managing TC during aneurysm coiling, several antiplatelet therapies with distinct profiles are available. Abciximab is commonly used as a rescue agent, though alternatives exist both within and outside the GP IIb/IIIa inhibitor class. Within this class, tirofiban and eptifibatide are frequently employed. Some studies suggest tirofiban may achieve higher rates of immediate recanalization (90.9% vs. 72.7% for abciximab in a small cohort) and potentially lower hemorrhage rates, although statistical significance varies (22). A meta-analysis further indicated that eptifibatide might achieve higher complete recanalization rates compared to abciximab or tirofiban (23). Outside this drug class, fibrinolytics like recombinant tissue plasminogen activator (rtPA) can be used, but carry higher hemorrhage risks, including a 10% rebleed rate in one series (24). GP IIb/IIIa inhibitors are often preferred over fibrinolytics due to their safer profile. It is critical to differentiate these intraprocedural rescue therapies from prophylactic oral antiplatelets (e.g., aspirin, clopidogrel), which are administered preemptively before elective procedures to reduce baseline risk of thromboembolic events (25). Agent selection ultimately depends on clinical context, hemorrhage risk assessment, and institutional protocols.

To our knowledge, systematic post-interventional infarction assessments and IL quantifications using DWI-MRI were not carried out in previous studies. Previous studies generally relied on CT imaging for infarction assessments. Additional novel aspects of our study are the consistent subgroup analyses of elective interventions and acute interventions due to SAH and the long-term clinical follow-up through the recording of mRS scores.

Several limitations of this study should be acknowledged. The retrospective design and non-randomized abciximab application limit definitive assessment of its effectiveness and introduce potential selection bias. Comparison of patients with and without TC may not fully capture abciximab’s effectiveness. Varying disease severity, reflected by H&H scores and DCI prevalence, likely influenced the observed differences in group comparsions in SAH cases. These factors underscore the complexity of evaluating interventions across different severity levels and necessitate cautious interpretation of our findings. Further, patient inclusion was limited to those able to undergo MRI within 7 days post-intervention, potentially excluding more severe cases, patients with MRI contraindications, and those who continued care abroad, which may affect the representativeness of our cohort. IL was measured using a simplified 2D method rather than full volumetric assessment and the lack of standardized abciximab dosing complicates result evaluation and comparison with other studies. Importantly, the sample size of patients with ruptured aneurysms treated with abciximab was small (*n* = 18), which limits statistical power and the reliability of subgroup analyses. Reflecting a common challenge in the literature where similar studies also feature small cohorts, our findings should be considered hypothesis-generating and require validation in larger patient populations. The dosing of abciximab in our cohort was highly variable, reflecting both the individualized nature of clinical decision-making and the lack of standardized protocols in the current literature (9). This variability limits the ability to assess dose–response relationships and may affect the generalizability and interpretation of our safety and efficacy findings. Lastly, abciximab’s market withdrawal in some countries limits direct application, though our findings may be relevant to other glycoprotein-IIb/IIIa receptor antagonists pending further research. These limitations should be considered when interpreting the study’s findings and their generalizability.

## 5 Conclusion

Based on our findings, IA abciximab, appears to be a safe and effective method for the management of TC during ET of intracranial aneurysms. Importantly, we observed no additional bleeding events following abciximab administration, further supporting its safety profile in this context. However, its potential benefit in SAH patients requires confirmation through randomized controlled trials.

## Data Availability

The original contributions presented in the study are included in the article/supplementary material, further inquiries can be directed to the corresponding author.
